# Quantitative evaluation of human sperm viability using MTT assay: A laboratory study

**DOI:** 10.18502/ijrm.v13i11.7966

**Published:** 2020-11-22

**Authors:** Hamid Reza Momeni, Mohammad Hussein Abnosi, Najmeh Eskandari

**Affiliations:** Department of Biology, Faculty of Science, Arak University, Arak, Iran.

**Keywords:** Human sperm, Viability, MTT assay.

## Abstract

**Background:**

3-(4, 5-dimethylthiazol-2-yl)-2, 5-diphenyl tetrazolium bromide (MTT) assay which evaluates cellular mitochondrial activity is widely used for the assessment of cell proliferation and viability.

**Objective:**

This study was performed to assess human sperm viability using MTT assay.

**Materials and Methods:**

In this laboratory study, human-ejaculated semen samples (n = 56 from different donors) were used. The sperm viability was determined using quantitative MTT assay and the sperm motility was assessed according to World Health Organization guidelines. Sperm viability and the correlation between sperm viability and motility were analyzed.

**Results:**

Data revealed a marked positive correlation between MTT reduction rate and the percentage of viable spermatozoa. The Pearson's correlation coefficients also showed a significant correlation between sperm viability and motility.

**Conclusion:**

MTT assay which is based on mitochondrial functionality is a reliable method for evaluating human sperm viability and could be used as a diagnostic test for predicting sperm fertilization ability in clinical settings.

## 1. Introduction

Most clinical laboratories and infertility centers often analyze the physical parameters of sperm, including the concentration, motility, and morphology in the semen, to report the sperm quality for predicting the sperm fertilization ability. However, around 15% of infertile men have a normal spermiogram (1). Therefore, this routine semen analysis may be deemed as a weak predictor of sperm quality and does not address other crucial parameters of sperm such as sperm viability, which can provide valuable information about predicting sperm fertilization ability. Accordingly, several methods have been developed to evaluate sperm viability. Some of them are based on dye exclusion including eosin-nigrosin (E&N) and trypan blue (TB) staining. These tests are based on the permeability and impermeability of sperm membrane which distinguish dead and live spermatozoa, respectively (2, 3). Although E&N and TB staining are regarded as simple and inexpensive methods for staining sperm smears, they might be affected by different analysts. Flow cytometry enumerates viable sperm in a cell suspension in a rapid and reliable manner. However, this method requires expensive instruments that may not be easily available in most laboratories. Sperm viability can also be examined by estimation of sperm metabolism status. 3-(4,5-dimethylthiazol-2-yl)-2,5-diphenyl tetrazolium bromide (MTT) assay is a valid and sensitive method for evaluating the metabolic activity and mitochondrial functionality of cells. The assay relies on the conversion of MTT, a yellow water-soluble tetrazolium salt, to purple-colored insoluble formazan crystals. This reaction involves mitochondrial dehydrogenases. Thus, this colorimetric assay determines the mitochondrial activity as a marker for cell viability. This assessment method was first introduced by Mosmann for the evaluation of lymphocytes viability and proliferation (4). This method has also been used for the quantitative estimation of the viability of bovine (5) and equine sperm (6). Although Nasr-Esfahani and co-workers (7) used MTT assay for the assessment of human spermatozoa viability, they stained spermatozoa only with MTT for selecting viable sperm from a sperm population in order to inject it into the egg cytoplasm. The functionality of sperm mitochondria is a critical factor that determines sperm capacitation, motility, and fertilization ability (8) and could be considered as a key parameter for estimating sperm viability. However, little attention has been paid to this parameter in clinical laboratories as it is not a common part of human semen analysis. Therefore, this study discusses the efficacy of MTT assay as a reliable, simple, and rapid method for evaluating human sperm viability. This assessment which is based on the evaluation of sperm mitochondrial functionality might be useful as a diagnostic test for predicting sperm fertilization ability in clinical settings.

## 2. Materials and Methods

### Preparation of semen

In this laboratory study, human-ejaculated semen samples (n = 56 from different donors) obtained from Sina laboratory, Arak, Iran, were used. The samples were prepared following an intercourse between couples and collected into a sterile plastic container after an abstinence period of two-three days. The samples were transferred to a physiology laboratory under standard conditions. parameters including sperm count and sperm motility were determined according to the World Health Organization (WHO) guidelines (9), to obtain general information about the quality of the semen. High-quality samples were then washed thrice with a culture medium composed of Ham's F10 (Gibco, UK) + 25 mM 4-(2-hydroxyethyl)-1-piperazineethanesulfonic acid, and HEPES (Sigma, USA) and centrifuged at 2500 rpm for 10 min. At the end, equal volumes of the culture medium were added to the final palette. The sperm suspension was counted and 3×10${}^{6}$ spermatozoa were added to 1 ml of the medium. This suspension was then used for the evaluation of sperm viability.

### Assessment of sperm viability

To evaluate sperm viability, MTT assay was performed according to a method described by Mosmann (4). The MTT solution (Sigma, USA) was prepared as a 5 mg/ml stock solution in phosphate-buffered saline (pH 7.4), filtered (0.22 μm, Millipore), and kept for no more than two weeks in the dark at 4°C. Next, 10 μl of the MTT stock solution was warmed at 37°C and added to the sperm suspension in an Eppendorf tube. The tubes were then incubated at 37°C for 1 hr (5) and centrifuged at 10000 rpm for 10 min. Supernatant was removed and pallet was resuspended in 200 μl dimethyl sulfoxide (DMSO, Merck, Germany) to dissolve the purple formazan crystals. The tubes were then centrifuged at 4000 rpm for 4 min and the supernatant was transferred to a 96-well microplate. The optical density (OD) of each well was measured using an ELISA reader (SCO diagnostic, Germany) at 505 nm.

**Figure 1:**
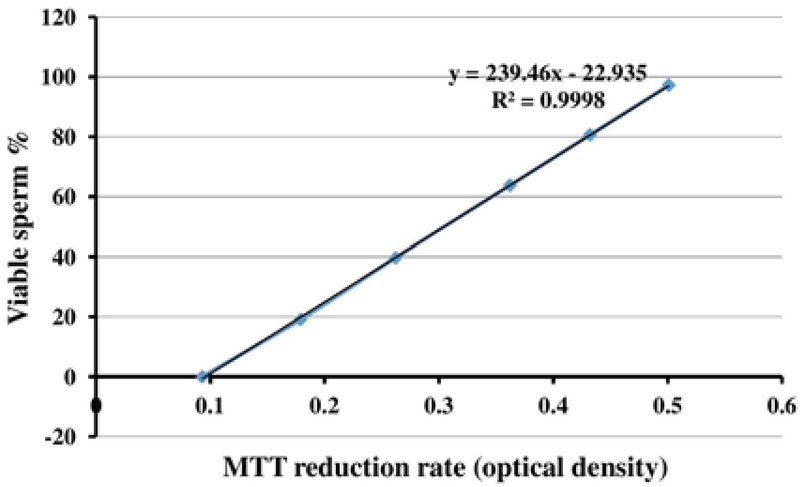
The standard curve presenting the correlation between 3-(4,5-dimethylthiazol-2-yl)-2,5-diphenyl tetrazolium bromide (MTT), reduction rate, and the percentage of human sperm viability.

To obtain the standard curve (Figure 1), the freeze-killed procedure was used as previously described (5). Accordingly, the sperm suspension (3×10${}^{6}$ high-quality spermatozoa) was divided into two fractions; while one fraction of the spermatozoa was kept at 37°C, the other fraction was killed by three cycles of freezing into liquid nitrogen and thawing at 37°C. Next, new samples were prepared by mixing spermatozoa at ratios of 10:0, 8:2, 6:4, 4:6, 2:8, and 0:10 (live spermatozoa: dead spermatozoa) (Table I). The samples were then analyzed by MTT assay and the correlation between MTT reduction rate and sperm viability was obtained using the following linear regression equation: Y = 239.4X-22.93, R${}^{2}$ = 0.994, where Y is the percentage of viable spermatozoa and X is the measured OD (Figure 1). This equation was used later for calculating the percentage of sperm viability in the 20 sperm samples.

**Table 1:** The correlation between 3-(4,5-dimethylthiazol-2-yl)-2,5-diphenyl tetrazolium bromide (MTT) reduction rate and the percentage of human sperm viability


** Number **	**Ratio of live: dead spermatozoa**	**Optical density (at 505 nm)**	**Sperm viability (%)**
** 1**	10:0	0.501 ± 0.02	97.20${}^{a}$ ± 4.80
** 2**	8:2	0.432 ± 0.04	80.58${}^{b}$ ± 9.62
** 3**	6:4	0.362 ± 0.03	63.74${}^{c}$ ± 7.22
** 4**	4:6	0.262 ± 0.05	39.68${}^{d}$ ± 12.00
** 5**	2:8	0.179 ± 0.01	19.70${}^{e}$ ± 1.21
** 6**	0:10	0.093 ± 0.01	0.00${}^{f}$ ± 0.00
Data are expressed as Mean ± SD, analyzed using ANOVA followed by Tukeyʼs test (n = 6 for each ratio, p < 0.05). Means with different superscripts differ significantly			

### Ethical consideration

The experiments conducted in the current study were approved by the ethical committee at Arak University of Medical Sciences.

### Statistical analysis

The results are expressed as Mean ± standard deviation (SD). One-way analysis of variance (ANOVA) followed by Tukeyʼs test was used to analyze the result on sperm viability. Pearson's correlation coefficient was used to evaluate the correlation between the percentage of sperm viability and sperm motility. P < 0.05 was considered significant.

## 3. Results

**Figure 2:**
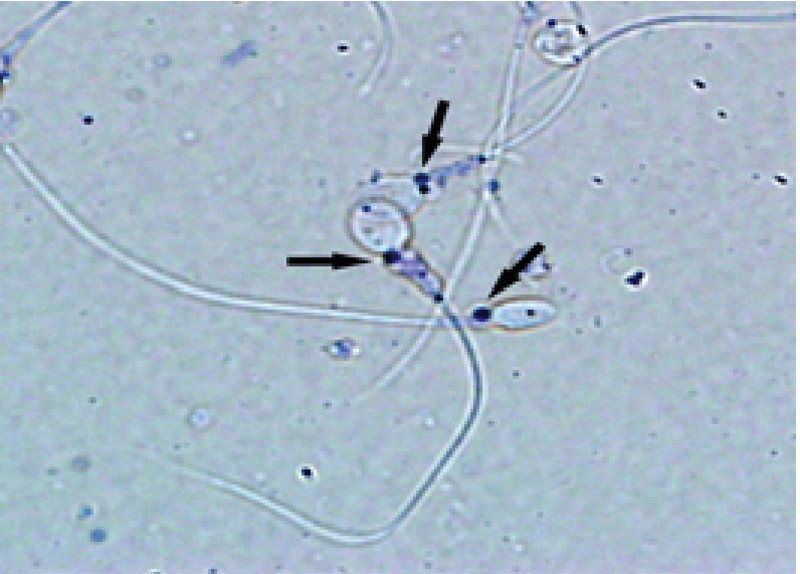
Formation of insoluble purple formazan granules (arrows) localized in the midpiece of viable human sperm stained by 3-(4,5-dimethylthiazol-2-yl)-2,5-diphenyl tetrazolium bromide (MTT) Magnification: 1000×.

Viable spermatozoa reduce MTT to MTT*-*formazan by their active mitochondrial dehydrogenases, where formazan crystals as insoluble purple color are localized in the midpiece of sperm (Figure 2).

The quantitative MTT assay showed a correlation between MTT reduction rate and the percentage of sperm viability in sperm samples containing different ratios of live and dead spermatozoa. Sperm viability was significantly (p < 0.001) increased by decreasing amounts of dead spermatozoa (Table I).

The standard curve shows a positive linear correlation between MTT reduction rate as a predictor and sperm viability as response variable. Using the linear regression equation (Y = 239.4X-22.935), the percentage of sperm viability in 20 human semen samples was calculated (Table II). Moreover, this table shows sperm motility of the samples. The Pearson's correlation coefficient analysis (r = 0.767) revealed a highly significant (p = 0.001) correlation between the percentage of sperm viability and sperm motility.

**Table 2:** Sperm viability as measured by 3-(4,5-dimethylthiazol-2-yl)-2,5-diphenyl tetrazolium bromide (MTT) assay and sperm motility in 20 human semen samples


**Sperm parameters (%)**																				
**Viability**	100	99	98	86	93	75	44	94	34	87	93	96	94	93	30	98	100	96	96	94
**Motility**	71	63	65	65	60	50	42	60	49	57	60	91	60	52	28	64	70	59	65	64

## 4. Discussion

In the present study, MTT assay was used to quantitatively assess human sperm viability. This assessment is widely used for estimating the viability and mitochondrial integrity in many cell types (10-13) including the sperm cell of several species (5, 6, 14). Since impaired sperm mitochondrial activity negatively affects sperm viability, it might attribute to male infertility. Therefore, this evaluation could be useful as diagnostic test for predicting sperm fertilization ability. For sperm viability assessment, MTT assay was used. In this method, the yellow tetrazolium dye, MTT, is reduced by the active mitochondrial enzyme succinate-dehydrogenase into insoluble purple formazan that accumulates within metabolically active cells but not the dead cells (4). The presence of purple formazan crystals in the midpiece of sperm showed that active mitochondria in spermatozoa were able to convert MTT to MTT formazan indicating that sperm is a live cell. Therefore, in this test, MTT reduction rate directly corresponds with the number of viable cells and mitochondrial activity (4). Accordingly, our results showed a high correlation between MTT reduction rate and the percentage of viable spermatozoa as it increased with a decrease in the amount of dead spermatozoa. MTT assay determines sperm viability rapidly. We showed an optimal time for MTT reduction for human sperm. After 1 hr incubation of sperm suspension with MTT solution, purple formazan was successfully formed and could be quickly extracted by DMSO. Our result is, therefore, in agreement with Aziz's report (5), who showed that the optimal time for MTT reduction by bovine spermatozoa was 1 hr. This observation was expected because sperm with rich mitochondria is a highly active cell and can quickly convert MTT, which shows that the reduction of MTT by spermatozoa could be faster than other cells (4). Moreover, MTT assay has been used more frequently compared to other viability assays such as eosin-nigrosin (15), TB (3) or hypo-osmotic swelling test (16), which assess plasma membrane integrity, but provide no information about mitochondrial activity or metabolic estate of sperm. Although this test is widely used to assess cell viability and proliferation (4), and to measure mitochondrial integrity, it should be born in mind that MTT reduction can also be a result of the activity of dehydrogenases outside the mitochondria (17). Nevertheless, MTT assay is still a convenient and inexpensive method for assessing cell viability in a variety of cells (10, 13) and tissues (18).

“Viability and motility are considered as the most important parameters of mature sperm which indicate its structural and functional quality to move toward an egg for successful fertilization” (19). Sperm motility could also be a predictor for sperm quality and fertilization ability (20). The viability of sperm samples obtained from 20 donors had a highly significant correlation with sperm motility. Since mitochondria are responsible for energy maintenance required for sperm motion, mitochondrial dysfunction may affect sperm motility leading to male infertility.

In conclusion, the present study introduces MTT assay as a quantitative assessment of human sperm viability and proposes this method as a diagnostic test for predicting sperm fertilization ability in clinical settings.

##  Acknowledgements

The authors would like to thank Dr. Mohammad Ali Daneshmand and Dr. Jalal Rezai, administers of Sina laboratory, Arak, Iran, for their wonderful collaboration.

##  Conflict of Interest

The authors declare that they have no conflict of interest.
